# Teaching of chronic pain management in a low- and middle-income setting: a needs assessment survey

**DOI:** 10.1097/PR9.0000000000000708

**Published:** 2019-01-21

**Authors:** Nomaqhawe Moyo, Farai Madzimbamuto

**Affiliations:** Departments of a Anaesthesia and Critical Care Medicine and; b Health Professions Education, University of Zimbabwe College of Health Sciences, Harare, Zimbabwe

**Keywords:** Pain, Chronic, Education, Low- and Middle-Income Countries, Curriculum

## Abstract

**Introduction:**

Pain is the most common reason for patients to see their physicians. For this reason, every physician should be able to diagnose and manage pain correctly.

**Objectives:**

The objective of this survey was to identify gaps in the teaching on chronic pain and its management in the current training programs at the University of Zimbabwe College of Health Sciences (UZCHS).

**Methods:**

A focused needs assessment using a self-administered questionnaire to collect data from participants was performed. A copy of the English Version of the International Association for the Study of Pain (IASP) curriculum on Pain for Medicine was attached for participants to refer to. Faculty and final-year postgraduate students were eligible. Questions were on the current teaching practice on chronic pain at the UZCHS.

**Results:**

Faculty members and postgraduates from 15 of 19 departments in the college participated in the study. Only 2 departments have written chronic pain teaching curriculum. Most faculty members, 68% had no knowledge of the IASP curriculum or its existence. Seventy-seven percent of the respondents were strongly dissatisfied with the current pain-related content, teaching or learning strategies in their programs. Most respondents suggested the need for the adoption of an interprofessional education learning strategy and adopting the IASP curriculum.

**Conclusion:**

The current teaching practice on chronic pain at the UZCHS is inadequate to prepare the health care professionals to independently care for chronic pain patients.

## 1. Introduction

Chronic pain affects people all over the world, including those living in low- and middle-income countries (LMICs) that have limited resources for health care services.^3,7^ There is a huge unmet need for chronic pain management across the globe. Chronic pain has become a major public health problem, and unrelieved pain is a significant public health concern.^2^ Wanderer in 2016 showed an infographic, which reviewed the geographic distribution of research focused on chronic pain in LMICs and described the most prevalent types of pain observed. Only 19 articles from 28 LMIC were found.^10^ This is a very small research output relative to the size of the population of people living in the LMIC. There is scanty data profiling the extent of the problem and what measures are being put in place to improve the care of chronic pain patients in the resource-limited setting. The International Association for the Study of Pain (IASP) reviewed data on pain education and management in low-resource countries fifteen years ago.^8^ There was no literature on pain education and curricula review in resource-limited settings. Despite the interventions introduced by the IASP, since then disparities still persist and resources are still needed.^8^ The lack of training in pain management for health care workers is the largest obstacle to good pain treatment in many countries. Every practicing physician is confronted with pain-related problems almost on a daily basis. Pain is the most common reason for patients to seek medical attention.^6^ For this reason, every health service provider should be able to recognise, assess, diagnose pain correctly, and institute treatment using simple treatment methods, algorithms, interventions, and be familiar with the referral network for interprofessional patient management.

Pain medicine is not taught as an independent study field during the training of health care providers both at undergraduate or postgraduate level in most medical schools.^2,3,11^ The subject of chronic pain remains an abstract subject with minimal cognitive and almost no psychomotor skills acquired during the process of medical training. Knowledge deficits among health care professionals are a major barrier to optimal pain management and a major contributing factor to the increasing number of people living with unrelieved chronic cancer and noncancer pain.^2^ Many medical practitioners do not have the confidence to independently manage chronic pain patients.

Pain medicine has now developed into an independent specialty, with its own fundamental core knowledge and competencies. This core knowledge can now be presented as part of a stand-alone module that can be tailored to undergraduate and postgraduate trainees.^1,4^ The objective of pain medicine education is to provide training to learners in which they will develop their knowledge, skills, and professional attitudes in the safe, comprehensive, compassionate, and confident management of patients in pain.^9^ Efforts to improve pain education in any medical training program should be based on a comprehensive understanding of how it is currently delivered and assessed.^2^ This needs assessment survey was performed to assess the current teaching practice on the subject of pain at the University of Zimbabwe College of Health Sciences (UZCHS).

## 2. Methods

### 2.1. Design

#### 2.1.1. Questionnaire survey

Ethics clearance was obtained from the Joint Research Ethics Committee for the University of Zimbabwe (JREC/414/16) and the study approved by the Medical Research Council of Zimbabwe (MRCZ/B/1239) before data collection was commenced.

The study population included the following:

(1) UZCHS faculty members in both the Basic Science and Clinical Teaching departments
(2) UZCHS final year Masters of Medicine students in the Clinical Medical departments.

A self-administered questionnaire created de novo was used to collect data for the survey from the faculty members. A different self-administered questionnaire created de novo was also used to collect data from postgraduate students. The data were collected from the September 1, 2016, to November 30, 2016. Questionnaires were distributed through the department chairperson offices. Twenty-eight of 40 questionnaires for faculty were returned and 27 of 34 final-year postgraduate students were returned.

The key questions asked included the following:

(1) Questions on the current teaching practice on the subject of chronic pain such as the content taught, the time spent teaching or learning on chronic pain, and its management;
(2) Teaching or learning strategies used to deliver chronic pain–related content in the curriculum;
(3) Assessment methods on the competencies on chronic pain theory and management;
(4) Knowledge of the IASP curriculum on Pain for Medicine;
(5) There was room for suggestions on ways to improve the current teaching and learning strategies on the specific subject of chronic pain.

A copy of the English Version of the IASP curriculum on Pain for Medicine (2012) was attached for participants to refer to as the reference standard for teaching on Pain.

The UZCHS is the biggest medical school in Zimbabwe. It has 19 clinical teaching departments, which include Anaesthesia and Critical Care, Clinical Pharmacology and Toxicology, Community Medicine, Dentistry, Haematology, Health Professions Education, Pathology, Medical laboratory science, Medicine, Nursing Sciences, Obstetrics and Gynaecology, Oncology, Ophthalmology and Optometry, Paediatrics, Pharmacy, Psychiatry, Physical Rehabilitation, Radiology, and Surgery. The college offers both undergraduate and postgraduate programs in the different department specialities. On average, there is an enrolment of 35 to 40 postgraduate students in the clinical medical departments each year.

## 3. Results

Faculty members and postgraduates from 15 of 19 clinical teaching departments in the college participated in the study. Radiology, Haematology, Medical laboratory Science, and Ophthalmology departments did not participate.

Only 2 departments have formal written chronic pain teaching curriculum namely Physiotherapy and Oncology. The physiotherapy undergraduate program is a 4-year taught course. The curriculum dedicates total 35 teaching hours for both the basic science and clinical application on pain. This covers both acute and chronic pain teaching. Oncology has a 4-year taught master's program. Their curriculum dedicates 70 teaching hours to the subject of pain. This includes acute, chronic, and palliative pain care. Undergraduates rotate in Oncology for 2 weeks and are not examined at exit of rotation. Both departments are aware of the existence of IASP guidelines; however, they have not adopted or adapted their content to the suggested guidelines. The pain content is delivered as part of the different medical conditions taught. There is no stand-alone pain module.

Most faculty members, 68%, had no knowledge of nor the existence of the IASP curriculum on Pain for Medicine. Faculty from Anaesthesia, Oncology, and Physiotherapy had knowledge of the existence of the IASP curriculum but had not adopted any part of the curriculum into their teaching.

None of the participating departments have adopted or adapted the IASP curriculum in their teaching.

Seventy-seven percent of the respondents were strongly dissatisfied with the current pain-related content taught, teaching, or learning strategies used in their respective programs of study. One hundred percent of the students were strongly dissatisfied with current teaching strategies.

Most respondents both faculty and students suggested the need for the adoption of an interprofessional education learning strategy using the IASP Pain curriculum as a reference guide, tailored to the local needs and resources. Most suggested a stand-alone pain module covering the basic science and clinical application on pain medicine in an interprofessional learning environment.

## 4. Discussion

Pain is the commonest presenting complaint to health care service providers.^4^ Many people are living with pain in the community. This has a huge toll on the patients, their families, and the society at large.^4,7^ All health care personnel should therefore be able to recognise, assess, and manage a patient presenting with pain. Literature, however, has shown that most practitioners pass out of their training programs without acquiring adequate skills to confidently and optimally manage pain, both acute and chronic.^2,6^ This has been shown to be a result of deficiencies in the medical curriculum, which have not been addressing the subject of pain comprehensively.^1,3^ In this study, only 2 departments of the 15, which participated in the survey had a formal written curriculum on pain, namely Oncology and Physiotherapy. This shows that there are no set standard teaching goals and learning strategies on the subject of pain in the college. Oncology and Physiotherapy generally deal with people living with chronic pain, and therefore, it is not surprising that they have formal curricula on the subject. However, their curricula have not adopted or adapted any of the IASP curricula on Pain for Medicine content. The subject on pain is taught randomly depending on the condition being managed, and there are no set objectives or goals on the content to be covered. There is no clear protected time set for teaching pain-related content in most of the departments. The use of interprofessional learning strategies to build on the interprofessional management of chronic pain patients is not evident in the departments which participated. Some departments, however, do teach on referral to other disciplines for a multidisciplinary patient management. The postgraduates had no clear knowledge of the expected content, knowledge, attitudes, and professional skills on chronic pain, which they were expected to have achieved on graduating from their programs.

Documented pain teaching in many European medical schools fall far short of what might be expected given the prevalence and public health burden of pain.^2,3^ Similarly, a study performed in Australia and New Zealand by Shipton et al.^9^ showed that despite the complexity of the topic on pain, most medical schools did not have well-documented or comprehensive pain curricula that are delivered and assessed using pedagogically sound approaches. The UZCHS similarly has no robust approach on the teaching and assessment on the topic of pain.

There is an urgent need to improve medical education systems to incorporate the teaching on the subject of pain medicine into the mainstream undergraduate and postgraduate curricula.^6^ Pain education is entering a new era of progress and impact as shown by the declaration of 2018 as the Global Year for Excellence in Pain Education by the IASP.

In an effort to address the inadequate teaching on the subject on chronic pain, the authors undertook a curriculum review and design process. An undergraduate curriculum on chronic pain medicine is being designed. The proposed curriculum is to be delivered as an elective module over a week. The long-term plan is to incorporate the module into the main UZCHS undergraduate medicine curriculum, which is currently undergoing a review process. The elective module is designed to deliver the basic core knowledge and theory of chronic pain, assessment, and basic treatment plans.^1^ Interprofessional learning strategies will be used to deliver the content to reinforce the importance of an interprofessional approach to chronic pain management.^5^ At the end of the module, the learners will be expected to be able to recognise, assess, diagnose, and draw up a comprehensive interprofessional treatment plan to manage a chronic pain patient. Common chronic noncancer conditions will be used as learning points, for example, chronic backache, knee osteoarthritis, and headache.

## 5. Conclusion

The current teaching practice on chronic pain at the UZCHS is inadequate to prepare the health care professionals to independently care for chronic pain patients.

The postgraduate students in the clinical departments pass out of the programs as Specialists in their fields of training; however, they are not equipped with adequate basic knowledge required to provide care for chronic pain patients.

There is very little protected time in the curriculum dedicated to the teaching on the subject of pain and chronic pain in particular.

A significant proportion of the faculty members and students have no knowledge of the existence of the IASP curricula.

In conclusion, there is a need to design a teaching module, which introduces a formal comprehensive approach to the teaching on chronic pain and its management to both undergraduate and postgraduate students at the UZCHS.

## Disclosures

The authors have no conflict of interest to declare.
